# Fora fuelling the discovery of fortified dietary supplements – An exploratory study directed at monitoring the internet for contaminated food supplements based on the reported effects of their users

**DOI:** 10.1371/journal.pone.0215858

**Published:** 2019-05-15

**Authors:** Nelleke H. J. Oostdijk, Mattijs S. Lambooij, Peter Beinema, Albert Wong, Florian A. Kunneman, Peter H. J. Keizers

**Affiliations:** 1 Radboud University Nijmegen, Department of Language and Speech, Nijmegen, the Netherlands; 2 National Institute for Public Health and the Environment, Bilthoven, the Netherlands; 3 Polderlink bv, Malden, the Netherlands; Universita degli Studi della Campania Luigi Vanvitelli, ITALY

## Abstract

Dietary supplements are products that are widely used for instance as energisers or to lose weight. There have been cases reported where undeclared ingredients present in such supplements have caused adverse effects on the health of the user. As there are many different products to choose from, it seems impossible to predict which might contain harmful components and to ban them from the market. Nonetheless, the use of dietary supplements and the experiences of users are shared in online discussions. We describe the development of a search engine to retrieve products associated with certain effects. Upon application we were able to retrieve a list of dietary supplements that are repeatedly associated with excessive effects by users on public fora. The top of the list contains supplements that have previously been banned because they contained undeclared harmful components. The use of the search engine as described here is a powerful method for making a risk-based selection of dietary supplements which can then be analysed for the presence of illegal or other unwanted components.

## Introduction

Dietary supplements are products that increase the intake of components whose supply via the daily diet is inadequate. Such products may contain bioactive substances, i.e. components which have a physiological activity or function. Their full composition must, however, always be declared and products must be safe for use and not jeopardise people’s health [1, 2]. Nevertheless, many examples exist of undeclared bioactive substances in dietary supplements being involved in causing adverse effects, leading to serious harm such as cardiac arrest and haemorrhagic stroke [3, 4]. Specifically, workout and weight-loss products are often spiked with stimulants making the products (more) effective, which seems to be part of a marketing strategy aimed at reeling in customers [5]. As the product label does not list these substances, the adverse effects they may cause are not readily recognised and likely to be underreported.

With a global dietary supplements market size valued at USD 133 billion in 2016 [6], and new products being launched on a daily basis, it seems impossible to identify products that could be harmful to the user. Elaborate chemical analysis of a product, in a search for unknown components is normally only performed after a product has been obtained from hospitalised victims [7]. Thus, the dietary supplements “Dexaprine”, “Iomax” and “Craze” were examined in the Dutch National Institute for Public Health and the Environment laboratory after users had experienced severe adverse effects. These supplements were found to be tainted with undeclared active substances which caused the effects [4, 8, 9].

In discussions on online fora, participants discuss the use of all kinds of dietary products that may be used to enhance physical performance or well-being. Thus, among practitioners of fitness and body building, it appears to be common knowledge what products to use to what effect; there is also a degree of awareness of the potential risks involved. Posts by users sharing the experiences that they have had with the use of particular products form a rich source of information. The internet can also provide access to the latest information especially in a dynamic market where new products keep emerging.

Where reported effects are beyond what could reasonably be expected, or where people suffer from health problems following the use of such products, this may be taken as an indication that the product is suspect and in need of further investigation.

When we searched the internet for information about the composition and use of the three supplements mentioned above using the Google search engine, they appeared to feature in multiple forum posts. This led us to the idea of conducting an exploratory study to investigate whether it would be feasible to develop a method of monitoring internet fora for mentions of dietary supplements that give rise to side effects or effects that sound too good to be true. Eventually, the composition of such products could be analysed for the presence of (undeclared) active ingredients. If the results come back positive, the authorities can then take appropriate action, for example, by banning a product from the market and issuing public warnings before the casualties themselves urge the government to take the same measures.

The present study is directed at mining Dutch forum posts of users of dietary supplements. The authors of the posts are mostly laymen without any medical background or training who exchange information on what products to use if they want to increase their body mass, lose weight, gain muscle, feel more energetic, etc.

Previous studies on user-reported effects have investigated adverse drug effects, while only a few studies have focused on dietary supplements [10]. Moreover, studies more generally have used data from clinical reports to this end rather than social media data. Notable exceptions are, for example [11–14]. The majority of studies originate from the US and involve English language datasets. We are not aware of any studies targeting Dutch data which might have been helpful in our method development. They report on their experiences using the everyday language commonly used on social media. As we were targeting dietary supplements used by Dutch users, we restricted ourselves to Dutch fora and, more specifically, to threads where the availability and use of dietary products were recurrent topics of discussion.

Approaches targeting English (U.S.) data may benefit from the various dictionaries, thesauri and databases that are available and from which product names and their related effects might be derived. Thus, the American National Institutes of Health has published the Dietary Supplement Label Database (DSLD; https://www.dsld.nlm.nih.gov) which includes ‘full label derived information from dietary supplement products marketed in the U.S.’, the Dietary Supplement Ingredient Database (DSID; http://catalog.data.gov/dataset/dietary-supplement-ingredient-database), and the ODS Dictionary, an online dictionary of dietary supplement terms (https://ods.od.nih.gov). The SIDER (‘Side Effect Resource’, http://sideeffects.embl.de) is a database of drugs and adverse drug reactions. There are barely any such resources available for the Dutch language. The Dutch Dietary Supplement Database (NES), listing the composition of 2,200 supplements often used in the Netherlands in 2011 appears to be outdated [15]. Other available resources are directed at health professionals and use medical terminology which is unlikely to be used in online fora. Examples of these are the Lareb database [16], maintained by the Netherlands Pharmacovigilance Centre Lareb (https://www.lareb.nl) which can be used to search for adverse reactions to approved drugs, and the Gecommentarieerd geneesmiddelenrepertorium, (Commented medicines repertory) published by the Belgian Centre for Pharmacotherapeutic Information (BCFI; http://www.bcfi.be/nl), which lists information pertaining to the medicines available in Belgium, including the side-effects or adverse effects that they may have [17]. The open language resources available for Dutch, such as the OpenTaal lexicon (https://www.opentaal.org) or Open Dutch Wordnet (ODWN; http://www.cltl.nl/projects/current-projects/opensourcewordnet/), are directed at standard Dutch, as found, for example, in published written texts. These are not particularly suited for handling social media data. In addition, these resources do not cover, for example, the specific product names of dietary products.

In the study presented here, we investigated whether social media mining could be used for the early detection of suspicious dietary supplements. We developed a method and incorporated it in a customised search engine that can be used to retrieve relevant text passages in posts published on online discussion fora. Relevant passages in this context are those in which products or product names are mentioned alongside some (excessive or undesired) effects. Results can be aggregated and a ranked list of dietary supplements created which can be used as a risk-based model to assess possibly harmful products.

## Materials and methods

### Data collection and preparation

**Fig 1:**
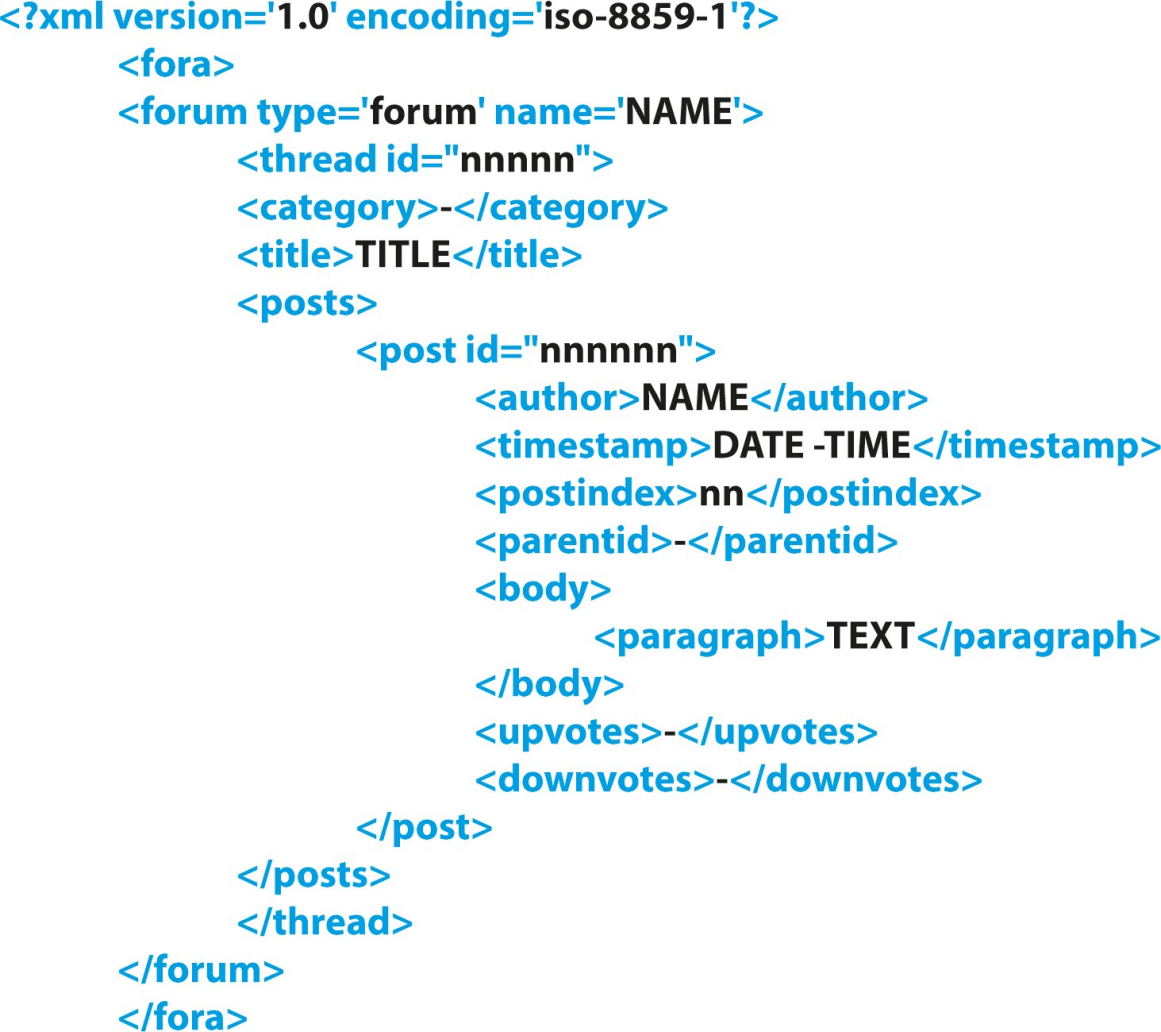
XML format used.

Relevant fora were identified using a number of keywords including *body building* and *fitness* (both English loanwords in Dutch), but also the names of some known tainted products. The fora that were used are listed in Table 1. Wget (version 1.19, GNU Operating system) was used to scrape the fora and the data obtained were converted to an XML format (Fig 1). With all posts, we retained information about the forum type, the thread the post was part of, title, author of the post, timestamp, post id, and post index. Where the data was found not to conform to the specified format, data clean-up was performed. The amount of work needed varied per forum. Corrections mostly involved incomplete or otherwise incorrect markup and the representation of special, non-standard ASCII, characters. Additional paragraph markers were introduced by replacing blank lines in the original by paragraph markers, so as to allow for the retrieval of shorter text passages from the occasionally rather lengthy posts. We decided on the paragraph as the unit for showing the output because paragraphs are relatively self-contained. Moreover, paragraphs were expected to provide sufficient context so that the user would be able to interpret the results of a search.

**Table 1:** List of the forum data used in this study.

Forum and URL	Start/end dates	# Posts	# Paragraphs
Biggerbody http://forum.bodybuilding.nl/	31 March 2002 to 17 July 2017	567	51,947
Body-fitness http://forum.body-fitness.nl/	2 November 2002 to 23 June 2016	28,334	1,164,118
Bodynet, thread Supplementen http://forum.bodynet.nl/	26 June 2002 to 21 June 2016	43,453	71,445
Bodynet, thread Afslanken http://forum.bodynet.nl/	26 August 2002 to 21 June 2016	2,064	62,688

To protect the privacy of forum users, data was stored for the time of the project and was only accessible for the researchers via a secured disk. We complied with the terms of service of the forum websites.

Although the fora were Dutch fora and the posts predominantly used the Dutch language, a fair proportion also included English texts. Upon closer inspection we found that English typically occurred with citations of published scientific articles that were brought to bear in support of something the author of a post was putting forward. As the English texts did not hamper our method, we decided not to remove them from our dataset. Part of the data was used for development, while a subset was kept apart for evaluation purposes. The composition of the datasets is shown in Table 1.

### Filtering

Filtering was done on the basis of term lists. Two lists were used: one for products and one for effects. Matching paragraphs should contain at least one item from each list. The lists were compiled by hand, using the data in our development set as well as whatever information we could find on the internet (published, for example, by online web shops selling dietary products).

#### Products list

To populate the products list there were no previously compiled lists that we could build on and the products mentioned on the sites of online web shops yielded too broad a range. We therefore decided to extract product names from the data we had scraped from the fora. Here we benefitted from the presence of the cited journal texts, as these used accurate spelling and capitalisation, and importantly, the registered trademark and trademark symbols. Using the Unix utility “grep” we extracted all the occurrences of items which were immediately followed by one of these symbols. This resulted in a first list of brand names and product names which, after inspection and clean up, was used as a seed list to extract further items. Thus, a number of patterns were specified for extracting additional product or brand names; for example ‘X van BRAND’, ‘X (BRAND)’, ‘X (van BRAND)’, ‘BRAND X’, and ‘PRODUCT van Y’ where BRAND and PRODUCT represented specific brand names and product names respectively, and X and Y represented the string to be matched (likely to be a product name or brand name respectively). X and Y would then be defined in terms of a regular expression. Thus, given that “Gaspari” is a brand name, the pattern ‘X van Gaspari’ yields matches where X is a likely product name and indeed, among the results we found “creatine”, “Halodrol”, “Mitotropin” and “Superpomp250”. In a similar fashion, knowing that “creatine” is a product name, the pattern ‘creatine van Y’ could be used to extract brand names, for example “Stacker”, “Gaspari” and “Bodylab”. Extracting the names from the data had the additional advantage that spelling variations and short forms of names were obtained as well.

#### Effects list

The effects list was built up from a vocabulary list of word n-grams and a rule- component. The word n-grams were mostly word unigrams (i.e. single words); only a small number of bigrams (i.e. two consecutive words, for instance a noun plus preposition) were included. With each item, the part-of-speech and semantic information was associated. This information was used by the rule-component in order to describe how the word n-grams listed in the vocabulary list could combine to form larger strings encompassing multiple words. For example, the vocabulary list included nouns like *diarree* (diarrhoea), *haaruitval* (hair loss) and *krachttoename* (increase in strength), and adjectives such as *kortademig* (shortness of breath) and *bloedverdunnend* (blood thinning) which, on their own, already denoted a specific effect. Other nouns like *effect* (effect), *toename* (increase), and *werking* (function) were semantically too vague to be used effectively in the search, as they can be used in a wide range of contexts. Therefore, having rules that specify which word n-grams can be combined, permits the specification of semantically meaningful strings. Where an adjective like *misselijkmakend* (nauseating) can be used in many contexts, in combination with a noun like *effect* it yields a useful term (*misselijkmakend effect*) for the search. Apart from nouns and adjectives, the vocabulary list also included adverbs, prepositions, verbs, and quantifiers, which made the identification of strings such as *lever aantasten* (affect liver), and *vocht vasthoud* (retain fluid) possible. Here it should be noted that with the adjectives and adverbs we also included sentiment words like *fenomenale* (phenomenal) and *dramatisch* (dramatic). Initially we created a separate vocabulary list of sentiment words and applied it independently of the effects list. This did not prove to be very effective as we would retrieve many irrelevant instances, where the sentiment was related to something other than a supplement. When we decided to include the sentiment words in the vocabulary list for the effects and allow the rules to force them to occur in combination with other items in the effects list, this yielded instances that were relevant such as *dramatisch afgevallen* (dramatic weight loss), *enorm opgefokt* (enormously stressed), and *fenomenale toename* (phenomenal increase).

As we were dealing with free text and there are many ways of expressing more or less the same information content, we introduced type labels that could be associated with effects strings. For example, based on the vocabulary list and the rules, we could match strings like *kan niet slapen* (cannot sleep), *‘s nachts wakker lig* (lie awake at night), and *last van slapeloosheid* (suffer from insomnia). Each of these strings indicated an effect on an individual’s ability to sleep. By associating a common type label, it became possible to aggregate the results in report classes. As we wanted to avoid the bias associated with suggesting that the labels might be indicative of some medical diagnosis, we chose labels that mostly referred to organs or other parts of the human body which were apparently involved. Some examples of the type labels used are ‘maag, lever en darmen’ (stomach, liver and intestines), ‘hart en vaatstelsel’ (heart and vascular system), and ‘longen en luchtwegen’ (lungs and respiratory system). Where it proved impossible to associate a particular type label, the label ‘unspecified’ was used.

## Inspection of the results obtained

The overall aim of our method was to extract those paragraphs from forum posts which mentioned the effects associated with the use of dietary supplements. The human expert then has the opportunity to inspect the results.

**Fig 2:**
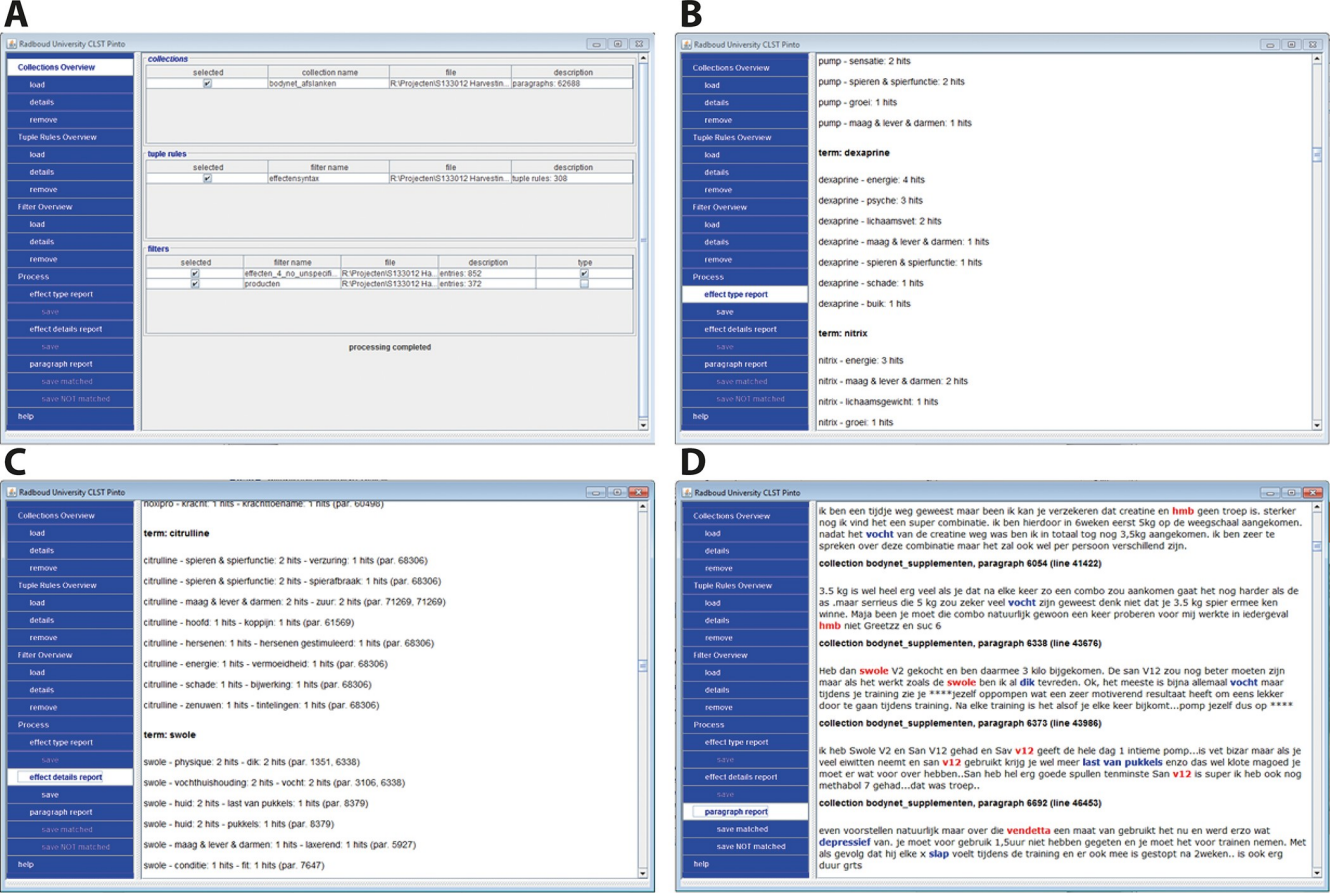
Interface of Pinto, displaying the overview of datasets, rules and filters (A), the results of a search in the effect type report (B), the effect details report (C), and the paragraph report (D).

To support the extraction process and facilitate the inspection of the results, the method was incorporated in Pinto, a customised search engine, operated by an interface depicted in Fig 2. Pinto (version 1.5.1, 2018) is a Java application created for the Centre for Language and Speech Technology of Radboud University. The Pinto software was made available for this project on a non-exclusive base and is made available through GitHub: https://github.com/centre-for-language-speech-technology/pinto. The core search engine used by Pinto is proprietary software made by Polderlink bv.

Pinto puts the human expert in control. Search using Pinto follows three steps: (1) selection and ingestion of data, i.e. forum posts, (2) processing, i.e. filtering, and (3) inspection of the results. In the first step, the user can select which of the collected datasets she wants to use from a list of available data sets which are presented in the form of a menu. There is also the possibility of opening new datasets or removing them. In the second step, the user selects which lists and rules sets to use for processing. A user can import new lists and rules sets or remove any of those available. New datasets, new lists or rules sets, whether these be created from scratch or by adapting existing data files, must be created outside of Pinto. Pinto enables selection and deselection, and also inclusion or removal from the menu, but does not support editing. Upon selection of a dataset, list or rules set, the user can ask for details. After the user has made her selection of dataset(s), lists and rules set to be used, she can start the processing. The combination of several filters, with the tuple rules, retrieves combinations of the filters only, within text paragraphs. Upon completion of the retrieval process the interface displays the results at three aggregation levels with increasing detail (Fig 2). At the first level, in the effect type report, the number of hits per category per product name are displayed. At the second level, in the effect details report, the paragraph numbers are added and at the third level, in the paragraph report, the full paragraphs are shown, with the found products in red and the effects in blue.

## Results

### Validation of the application

The performance of Pinto in retrieving the required information was initially assessed by comparing its output with that of an expert. To this end we retrieved all text paragraphs that mentioned the product name “RPM” from the thread “supplementen” on the Bodynet forum. These paragraphs (61 in total) were presented to a human expert, who was asked to mark all the effects (not the effect types) that were associated with “RPM”. The expert, who was the last author of this study, has a background in pharmacochemistry. We also used Pinto to identify those paragraphs in which “RPM” and one or more terms from the effects list were found to co-occur. Upon comparison of the two results (paragraphs containing effects marked by the expert as associated with “RPM” vs. paragraphs identified by Pinto), we found a precision of 0.74, a recall of 0.81 and an overall accuracy of 0.84. This first evaluation was primarily intended to obtain feedback as to the efficacy of the method we had implemented and was directed towards the quality of the results. As the number of mentions of “RPM” was limited, this set lent itself for full annotation by an expert which, in turn, made it possible to establish the level of recall obtained by Pinto. Efficiency in processing (expert vs. Pinto) was not considered to be an issue as it was evident that Pinto easily outperforms any human.

What we observed in terms of (qualitative) differences in effects marked by the expert as relevant vs. what Pinto found, or did not find, was that Pinto executed the task of retrieving all co-occurrences of the product name and one or more effects systematically and consistently, but without any understanding of the context. Therefore, as was to be expected, Pinto had a problem with negations and with cases where, in one and the same paragraph, more than one product was mentioned (as it had no way of knowing which product was referred to when an effect was reported). Thus Pinto incorrectly identified example i, see below, as a relevant paragraph as it contains a mention of “RPM” and the effects “meer kracht”, “explosieve kracht” and “meer uithoudingsvermogen”, while the effects are in fact associated with “creatine”; moreover, rather than “meer uithoudingsvermogen” “niet veel meer uithoudingsvermogen” should be considered. This is not to say that the expert was always correct. We have seen instances where, apparently, the expert was distracted, as for instance in example i, where ‘explosieve kracht’ was marked as an effect (of “RPM”), or example ii, where the effects mentioned were primarily attributed to the use of “Drive” rather than “RPM”. Where the expert clearly outperformed Pinto was in the recognition of sometimes quite creative expressions denoting some effect, such as we see in example iii; “strak als een bos uien in de sportschool staan” (find yourself in the gym as tight as a bunch of onions). Such expressions are invented on the fly and thus are not entered into any term lists.

- Creatine geeft mij vooral meer kracht, (zo’n 10% van mijn rpm erbij) explosieve kracht, niet veel meer uithoudingsvermogen. (Creatine gives me mostly more strength, (approx. an additional 10% of my rpm) explosive strength, not much more stamina.)
- Drive had ik een tijdje gecombineerd met RPM, maar vooral van de drive kreeg ik echt last van mijn maag. Boeren die echt ONGELOVELIJK stonken, vervelend stekend gevoel in mijn maag enz. (I had combined Drive for a while with RPM, but Drive, in particular, caused stomach aches. Burps that stank INCREDIBLY, nasty stinging sensation in my stomach, etc.)
- Weet niet hoe gevoelig je voor rpm bent, maar het lijkt mij dat je dan strak als een bos uien in de sportschool staat. (Don’t know how sensitive you are to rpm, but it seems to me that you would find yourself in the gym as tight as a bunch of onions.)

A second performance test was directed at establishing the difficulty of the task. Here we wanted to find out the extent to which humans were capable of identifying the effects reported and associated with particular products, and how much humans would agree between them. In addition we also looked at how the human judgements compared to Pinto results. We had two students (one with a background in Health Sciences and the other in Communication Studies) and an expert (a molecular scientist from the National Institute for Public Health and the Environment) annotate 480 paragraphs of text, in which one or more of five selected products were discussed. The annotators marked those parts of text that they found indicated any positive or negative effects and these selections were compared to the Pinto output for these same paragraphs. A total of 35.3% of the Pinto output was found relevant by all the annotators, 50.9% was found relevant by two annotators and 58.1% was found relevant by one annotator. This indicates that Pinto is successful in retrieving relevant passages, but also that it seems to be less stringent than the annotators. The stringency is tuneable by changing the effects filter. If less specific terms are removed from the filter, a lower number of hits is retrieved. A high score was not the goal for the performance test, but for the eventual use of Pinto in retrieving suspect supplements, less specific terms were removed. Therefore, in later instantiations of Pinto the effect strings labelled “unspecified” were opted out in the analysis for suspect supplements. When we compared the output of the three annotators and calculated the inter-annotator agreement, this yielded a Cohen’s kappa value of 0.69 between the students, 0.65 between the expert and student 1, and 0.71 between the expert and student 2. This indicates that there was considerable overlap in the marked parts of text and that no specific expertise seems to be required to recognise effects of interest.

### Output from a forum

**Fig 3:**
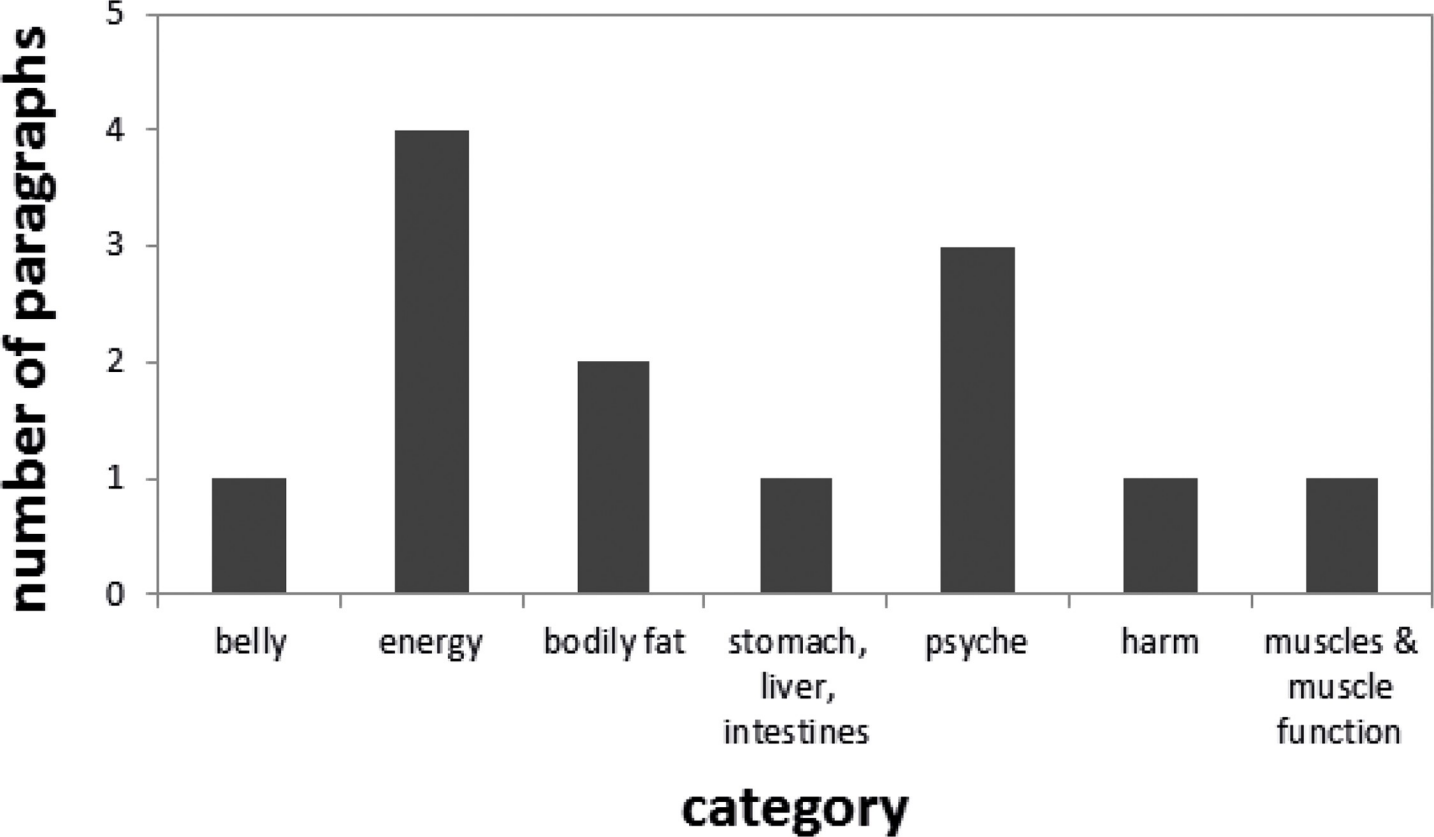
Number of positive paragraphs retrieved from the forum “Bodynet”, more specifically the thread “Supplementen”, for the dietary supplement Dexaprine, displayed per category (effect type).

The Google search engine was used to find public fora where dietary supplements were a topic of discussion. Names of dietary supplements banned from the market because of their contamination with undeclared ingredients and terms such as “forum” and “voedingssupplement” were used to find the fora. The fora selected (Table 1) were publicly available, had been in use for the last two years and contained more than 100 posts. Screening of the thread “Supplementen” from the forum”Bodynet” with Pinto on product names and effects, yielded 539 hits on 77 products in 28 categories (effect types). To try and rank the 77 products, the number of hits they had was taken as an indicator of a higher probability of strong effects. The products “BCAA”, “Crack”, “HMB”, “No-xplode”, “Rush”, “Tribulus” and “Vitargo” scored more than 20 hits. “Rush” had the highest score of 48 hits. The product “Dexaprine”, known to contain a cocktail of undeclared stimulating active substances, was found in the list of products with 13 hits, see Fig 3. “Dexaprine” scored in the categories “belly” (1), “energy” (4), “body fat” (2), “digestive tract” (1), “psyche” (3), “harm” (1) and “muscles” (1). “Rush” scored in all these categories as well, except for “belly” and “harm”, and in eight additional categories. The category “harm” could be key in indicating supplements causing adverse effects. Searching the dataset using the category “harm” as the only effect filter, yielded 18 paragraphs on 11 products. Upon closer inspection it appeared that the filtered term categorised as “harm”, was actually often used to report the absence of side effects. Therefore, additional categories seem useful to retrieve products of concern. The product “Dexaprine” scored high on “energy” and “psyche” as well, two categories which are not necessarily linked and could perhaps be a combination predictive for “harm”. Energising supplements having an effect on the category “psyche” could be a red flag. When searching specifically for supplements known to have caused adverse effects, we found that the dataset contained three paragraphs mentioning “Iomax”, none on “Jacked Power” and 33 on “Dexaprine”. Of these 33, 13 contained an effect in the listed categories and hence were retrieved by Pinto.

**Fig 4:**
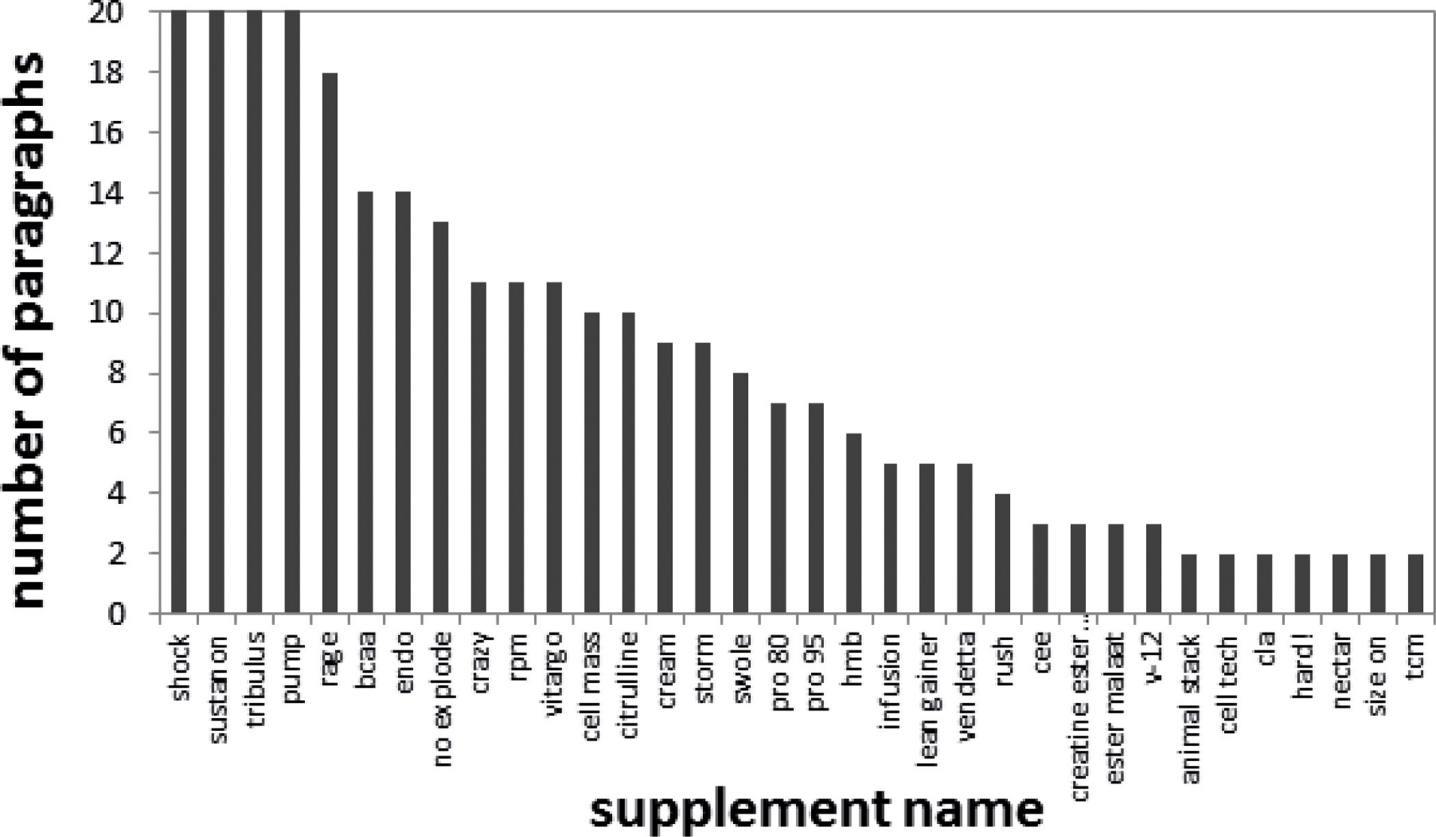
Results from searching the “Body-fitness” forum using Pinto, a products filter and an effects filter. The number of paragraphs in which the product was mentioned together with an effect was plotted per dietary supplement product name. A maximum of 20 paragraphs was plotted and only supplements with more than 1 hit were displayed.

Screening the thread “Afslanken” on the forum “Bodynet”, yielded 36 hits on 12 products. Most of the retrieved products were the same as those found in “Bodynet Supplementen”. Screening of the forum “Body-fitness” yielded 503 hits on 54 products, see Fig 4. Products with more than ten hits were “Shock”, “Sustanon”, “Tribulus”, “Pump”, “Rage”, “BCAA”, “Endo”, “No-explode”, “Crazy”, “RPM” and “Vitargo”. There was considerable overlap between the products discussed here and on the two “Bodynet” fora. Screening of the forum “Biggerbody”, yielded 15 hits on 6 products; “Shock”, “Sustanon”, “Rush”, “Endo”, “Dexaprine” and “Jack3D”. When looking at the paragraphs mentioning the products, most of these hits appear to be false positives. The words “shock”, “rush” and “endo” were not used as product names. “Dexaprine” and “Jack3D” were mentioned as examples of supplements rather than reported experience. “Sustanon” is a known anabolic steroid containing product and therefore not a target of this study.

## Discussion

Even though they can give rise to significant physiological effect, dietary supplements are not under the strict control that, for instance, medicines are. Independent testing of supplement composition or other quality hallmarks is performed on a risk basis and most of the time only after they have been associated with serious harm. We wondered if we could somehow detect suspect supplements among the vast offer of products available, using publicly available information. Eventually a shortlist of suspect supplements could be subjected to chemical analysis before serious harm is reported, and thus offer a tailored risk-based model of analysis.

While we were interested in any dietary supplement reported to cause an adverse effect, we often could not distinguish between what might have been an intended effect or a side-effect, let alone an adverse effect. For example, losing weight is what one would expect of a slimming product, but it might also occur as a side-effect of another type of product. In addition, even with slimming products, losing too much weight or losing weight too fast may point to something being amiss. This need not be related to the product though, as effects may well be explained by other factors. For example, effects may arise only when the supplement is being used in (too) high doses or inappropriately, or by people with particular health conditions. In this study, therefore, we did not differentiate between the different types of dietary supplements, nor did we distinguish between effects and side-effects. An effect is taken to be (the result of) any change in state or condition whether this be intended or brought about unintentionally.

It is the duty of the National Institute for Public Health and the Environment to protect public health and by developing Pinto we have tried to reach this goal by exploring text mining techniques. We have focussed on Dutch datasets corresponding to the legal tasks of the institute and the Inspectorates involved in enforcement. Given a collection of Dutch forum posts, a list of products/product names and a list of “effects” our method should be able to extract those passages in posts where a product(name) co-occurs with one or more effects. The results should be presented to an expert for further inspection. The method involves the following steps: (1) data collection and preparation, (2) filtering, and (3) inspection of the results.

The programme Pinto was developed and successfully applied to retrieve dietary supplements associated with certain effects. The fact that “Dexaprine” is found on several fora with a significant number of hits is indicative that this approach could be a successful one. Nevertheless, it remains unclear whether many hits should give rise to more concerns, or that a single but specific post could be more alarming. The use of Pinto in practice over time may answer this question.

There are limitations to the study. Firstly, the work is confined to the Dutch experience. Second, the created list of product names will never be complete and new product names must be added to the list on a regular basis to avoid false negatives. More difficulties are created by the false positives that are picked up as product names may not be specific; “shock”, “rush” and “endo”, for instance, are used in meanings which are different from product names. “BCAA”, “citruline” and “HMB” refer to product types, whose composition varies between specific batches produced by specific manufacturers. Any reported effect, positive or negative may not be specific for the type of product or main component. A manual check of product name lists and retrieved positive paragraphs will be required. Nevertheless, the current approach does provide a list of products independent of manufacturer description or declaration, which are at the moment the hallmarks by which products would be selected for a risk-based analysis. This provides an opportunity for unexpected products to be picked up that may turn out to cause adverse effects.

Public fora were used as the source for individual experience with dietary supplements in this study. Similar to other social media, public fora may turn out to be a way of communication typical of its time and, in the coming years, lose the interest of their users and become outdated. Alternatively, the users may switch to other forms of social media to share experiences. This latter case would be a positive scenario because the information, at least, would still be available and text mining an option for analysis. This would require adaptation of the search engine though.

The search engine could be technically improved by linking spelling variants and alternative names of the same product. A time line could also be created to make visible the peaks in attention that specific products have. In this way, the effects of warnings and market withdrawal on the experience of the users could be monitored.

The search engine may find application in other domains. It is anticipated that, by changing the filters and input data, Pinto will be useful in monitoring the use of new psychoactive substances, dietary intake in general, and any other subjective decision-related use of products or services that is publicly shared.

## Conclusion

With the newly developed search engine we were able to retrieve, with high accuracy, a list of food supplements that have been repeatedly associated with strong effects by users. The top of the list contains supplements that have been banned because they contained undeclared harmful components. The use of the search engine, as described here, is a powerful method of making a risk-based selection of dietary supplements to be analysed for the presence of illegal or other unwanted components.
